# Breast cancer cells promote a notch-dependent mesenchymal phenotype in endothelial cells participating to a pro-tumoral niche

**DOI:** 10.1186/s12967-015-0386-3

**Published:** 2015-01-27

**Authors:** Pegah Ghiabi, Jie Jiang, Jennifer Pasquier, Mahtab Maleki, Nadine Abu-Kaoud, Najeeb Halabi, Bella S Guerrouahen, Shahin Rafii, Arash Rafii

**Affiliations:** Stem Cell and Microenvironment Laboratory, Weill Cornell Medical College in Qatar, Education City, Qatar Foundation, P.O. Box 24144, Doha, Qatar; Department of Genetic Medicine, Weill Cornell Medical College, New york city, NY USA

**Keywords:** Tumor microenvironment, Endothelial cells, Phenotypic plasticity, TGFβ & notch pathways, Pro-tumoral niche

## Abstract

**Background:**

Endothelial cells (ECs) are responsible for creating a tumor vascular niche as well as producing angiocrine factors. ECs demonstrate functional and phenotypic heterogeneity when located under different microenvironments. Here, we describe a tumor-stimulated mesenchymal phenotype in ECs and investigate its impact on tumor growth, stemness, and invasiveness.

**Methods:**

Xenograft tumor assay in NOD/SCID mice and confocal imaging were conducted to show the acquisition of mesenchymal phenotype in tumor-associated ECs *in vivo*. Immunocytochemistry, qPCR and flow cytometry techniques showed the appearance of mesenchymal traits in ECs after contact with breast tumor cell lines MDA-MB231 or MCF-7. Cell proliferation, cell migration, and sphere formation assays were applied to display the functional advantages of mesenchymal ECs in tumor growth, invasiveness, and enrichment of tumor initiating cells. qPCR and western blotting were used to investigate the mechanisms underlying EC mesenchymal transition.

**Results:**

Our results showed that co-injection of ECs and tumor cells in NOD/SCID mice significantly enhanced tumor growth *in vivo* with tumor-associated ECs expressing mesenchymal markers while maintaining their intrinsic endothelial trait. We also showed that a mesenchymal phenotype is possibly detectable in human neoplastic breast biopsies as well as ECs pre-exposed to tumor cells (ECs^Mes^) *in vitro*. The ECs^Mes^ acquired prolonged survival, increased migratory behavior and enhanced angiogenic properties. In return, ECs^Mes^ were capable of enhancing tumor survival and invasiveness. The mesenchymal phenotypes in ECs^Mes^ were the result of a contact-dependent transient phenomenon and reversed upon removal of the neoplastic contexture. We showed a synergistic role for TGFβ and notch pathways in this phenotypic change, as simultaneous inhibition of notch and TGFβ down-regulated Smad1/5 phosphorylation and Jag1^KD^ tumor cells were unable to initiate the process.

**Conclusions:**

Overall, our data proposed a crosstalk mechanism between tumor and microenvironment where tumor-stimulated mesenchymal modulation of ECs enhanced the constitution of a transient mesenchymal/endothelial niche leading to significant increase in tumor proliferation, stemness, and invasiveness. The possible involvement of notch and TGFβ pathways in the initiation of mesenchymal phenotype may propose new stromal targets.

**Electronic supplementary material:**

The online version of this article (doi:10.1186/s12967-015-0386-3) contains supplementary material, which is available to authorized users.

## Background

Endothelial cells (ECs) are the building blocks of the vascular system and are characterized as the single-cell layer of epithelium that forms the inner cell lining of blood vessels and lymphatics [1]. Vascular ECs were initially considered passive conduits for delivering oxygen and nutrients to all tissues [2,3]. The development of appropriate culturing systems for primary human organ-specific ECs provided the opportunity to identify EC heterogeneity in different organs as well as their functional properties under normal and pathological conditions [4-6]. Today, ECs have been implicated in several perfusion-independent processes including tissue regeneration, tumor growth and dormancy through secretion of angiocrine factors [7-14]. In addition, increasing evidence reveals that endothelial cellular identity is more plastic than previously thought [15]. This plasticity results in phenotypical and functional modifications under different contextual conditions. A characteristic example of such phenotypic modification is endothelial-to-mesenchymal transition (EndMT), during which ECs lose their endothelial phenotype and acquire mesenchymal traits [16-18]. EndMT is implicated in tumor progression through complex modulation of the tumor and its stroma [17]. It is likely that precise analysis of cellular transformation in tumor microenvironments will reveal subsets of additional cellular phenotypes that might be drug targets and/or biomarkers.

In this study, we aimed to investigate the role of tumor cells in promoting mesenchymal phenotype in ECs by setting up tumor-endothelial co-culture systems in the absence of serum or cytokine supplementations. We initially confirmed the induction of mesenchymal phenotype in Human Umbilical Vein Endothelial Cells (HUVECs) by breast tumor cells. Then, to overcome the barrier of endothelial sensitivity to starvation and tumor cell-induced cell death [19], we continued our work with the previously described E4-ECs (that we here refer to as ECs) [10,20-22]. ECs were produced through transfection of Primary Endothelial Cells (PECs) with adenoviral *E4ORF1* gene as described previously [21]. While this transfection provides a low Akt activation allowing endothelial survival in a serum and cytokine-free condition, it does not modify the endothelial phenotype as has been widely used [10,20,22]. Besides, activation of Akt in tumor endothelium has been previously reported [23] and our model might thus be more optimal to mimic the crosstalk between ECs and cancer cells *in vivo* without any background effect. Using breast cancer cells (BCCs), we showed that BCCs in co-culture with ECs stimulated transcriptomics modification of ECs partly represented by acquisition of mesenchymal phenotype. While a similar phenomenon (EndMT) has already been described in the developmental and pathological context, we were able to show that tumor cells were capable of stimulating mesenchymal phenotypes in ECs and the tumor-associated ECs retained their endothelial properties while gaining mesenchymal phenotypes. In addition, this transition was reversible and dependent on continuous contact between ECs and BCCs. Subsequently, we showed that the mesenchymal ECs were capable of constituting a pro-tumoral niche responsible for increasing BCC proliferation, mammary stem cell self-renewal, and pro-metastatic properties. Our results also suggest that tumor-promoted mesenchymal shift in ECs is regulated by Smad signaling through the synergistic stimulation of TGFβ and notch pathways.

## Methods

### Cell culture & reagents

Breast cancer cell lines MDA-MB231 (MDA-231), MCF-7, and HUVEC were purchased from American Type Culture Collection (ATCC, USA). GFP^+^ECs (ECs) were developed as described previously [21]. Human recombinant Jagged1 and TGFβ1 were obtained from R&D Systems and PeproTech, respectively. Υ-secretase inhibitors (GSI) and SB-431542 were purchased from Sigma (USA). Breast cancer cells (BCCs) were grown in DMEM/High glucose (HyClone, USA) supplemented with 10% FBS, L-glutamine, non-essential amino acids (NEAA), and penicillin/streptomycin in a humidified incubator with 5% CO_2_. ECs were grown in M199 growth medium (Gibco, USA) supplemented with 20% FBS, 20 ng/ml β-Endothelial Cell Growth Factor (βECG), 20 units/ml heparin and penicillin/streptomycin. The co-cultures were prepared by mixing one part BCCs with 10 parts GFP^+^ECs (1:10 ratio) and cells were grown in 1:1 ratio of DMEM/High and M199 media in the absence of serum and growth factors (complete starvation). Co-cultivation of BCCs and ECs was performed over 3–5 days under adherent condition.

### Sphere forming assay

Sphere forming assay was used to enrich mammary stem cells (mammospheres) as previously described by Dontu [24]. We slightly modified that protocol and co-cultured mammospheres with GFP^+^ECs at 1:10 ratio under non-adherent condition to obtain mammo-angiospheres. Mammo-angiospheres were therefore composed of both tumor and GFP^+^ endothelial colonies mingling together. Spheres were grown in a so-called “*3D* media” as described by Dontu and colleagues by using DMEM-F12 (HyClone, USA) supplemented with 2% B27, 20 ng/mL basic fibroblast growth factor (bFGF) and epidermal growth factor (EGF), and 5 μg/mL insulin. In order to prevent the formation of cellular aggregates, a highly viscose *3D* media was prepared by addition of 0.2% methylcellulose (Sigma, USA). Stem cell enrichment was evaluated by measuring the perimeter of mammospheres or angiospheres with NIH ImageJ 64 software or by quantifying the number of spheres. A GFP filter was used to distinguish angiospheres.

### Cell proliferation assay

MDA-231 or MCF-7 cells were co-cultured with GFP^+^ECs (1:10 ratio) under starvation and ECs survival was assessed at different intervals by trypsinization and repeated manual counting by hemacytometer. A GFP filter was used to distinguish the GFP^+^ECs from unstained BCCs. In this study, ECs that have been pre-exposed to BCCs are referred to as ECs^Mes^, whereas ECs^Norm^ are normal ECs with no prior contact with BCCs. To see the effect of ECs^Mes^ on BCC proliferation and survival, GFP^+^ECs were directly co-cultured with MDA-231 and MCF-7 cells for three to five days to obtain GFP^+^ECs^Mes^ prior to initiating a proliferation assay. Next, we started a proliferation assay with ECs^Mes^ while still growing with BCCs and newly established co-cultures of GFP^+^ECs^Norm^ and BCCs for seven more days under complete starvation. BCCs either in mixture with GFP^+^ECs^Norm^ or GFP^+^ECs^Mes^ were then counted by trypsinization and manual counting excluding ECs by GFP filter.

### Flow cytometry & cell sorting

Antibodies to human PE-CD31 (560983), AF647-VE-cadherin (561567), and fibronectin (FN1, 610077) were purchased from BD Biosciences (USA). AF633-F-actin (phalloidin, 22284) is a product of Invitrogen (USA), vimentin (5741) and α-SMA (ab5694) are from Cell Signaling Technologies and Abcam, respectively (USA). The secondary antibodies were purchased from Invitrogen. GFP^+^ECs were either cultured alone or co-cultured with BCCs. To stain ECs in mono or co-cultures, cells were initially trypsinized and washed with PBS. For labeling intracellular proteins, cells were initially fixed then permeabilized on ice in freshly prepared 3.7% paraformaldehyde and 0.1% TritonX-100 for 10 minutes/each prior to incubation with primary antibodies (permeabilization by TritonX-100 was not carried out for cell surface proteins). Briefly, cells were resuspended at 1 × 10^6^ cells/100 μL density in a staining buffer containing 5% FBS, 1% BSA, 0.2 mM EDTA in PBS. To enhance the specificity of staining, FcR blocking (Miltenyi Biotec, USA) was added at 5 μL/1 × 10^6^ cells prior to incubation with primary antibodies. Primary antibodies were then added according to the instructions provided by the manufacturers and incubation was done for 1 hour at 4°C. After washing, cells were stained with secondary antibodies for 30 minutes at 4°C followed by washing. Fluorescent light (FL) was quantified using Fluorescence Activated Cell Sorting (FACS) on a SORP FACSAria II (BD Biosciences), eGFP fluorescence was acquired using a 488 nm blue laser and 510/50 nm emission, Phycoerythrin fluorescence (PE) was acquired using a 498 nm blue laser and 575/26 nm emission. Alexa Fluor® 647 fluorescence was obtained with a 650 nm red laser and 660/20 nm emission, while Alexa Fluor® 633 was obtained with 633 nm red laser and 647 nm emission. The figures display the median of fluorescence intensity (MFI) relative to controls. Doublets were excluded by FSC-W × FSC-H and SSC-W × SSC-H analysis, and single stained channels were used to compensate. Fluorescent minus one was used for gating. 10,000-30,000 events were acquired per sample. Finally, data were processed with FACSDiva 6.3 software (BD Biosciences) or Summit 4.3 (Dako).

For sorting GFP^+^ECs, GFP fluorescence was acquired using 488 nm blue laser and 510/50 nm emission and sorting was done using purity masks [13]. For sorting BCCs, cancer cells were stained with a PE-conjugated dye called PKH26 (Sigma, USA) prior to co-culture and PE fluorescent was acquired using 496/566 nm blue laser and 576 nm emission to separate them from GFP^+^ECs. Control GFP^+^ECs or PKH^+^BCCs monocultures were processed and sorted to normalize the cellular stress caused by cell sorting.

### Wound healing assay

GFP^+^ECs and PKH26^+^BCCs were co-cultured under starvation for 3–5 days, and then the cells were sorted as described in the previous section. Sorted ECs or cancer cells were immediately plated and grown at 100% confluence in complete medium to recover overnight. Next, they were continued to culture under complete starvation for 6 hours to impede cellular growth before a wound healing assay was initiated [25]. Finally, the migration capability of cells to close the wound (scratch) was evaluated after 48 hours using NIH ImageJ 64 software.

### Tube forming assay

Growth factor reduced Matrigel (BD Biosciences, USA) was thawed at 4°C overnight, and added to each well of a 48-well plate (120 μL/well) and allowed to solidify for 30 minutes at 37°C. GFP^+^ECs were sorted from BCCs and immediately plated on matrigel at subconfluent density (2.5 × 10^4^ cells/well). The formation of capillary-like structures was examined with an inverted microscope after 24 hours and the number of capillary junctions was quantified by analyzing the digitized images.

### Immunocytochemistry

Antibodies against PE-CD31 (560983), AF647-VE-Cadherin (561567), and FN1 (610077), CD44 (555478) and desmin (550626) were purchased from BD Biosciences. F–actin (AF633-phalloidin, 22284) is a product of Invitrogen, whereas vimentin (5741) and α-SMA (ab5694) antibodies are products of Cell Signaling Technologies and Abcam, respectively. Anti-fade gold DAPI and secondary antibodies were purchased from Invitrogen. Cells were grown, stained, and imaged on glass chamber slides (Lab-Tek®). Briefly, the adherent cells were washed once with PBS and fixed in 3.7% formaldehyde, then permeabilized in 0.1% Triton X-100 for 20 minutes (no permeabilization required for cell surface proteins). After one wash, the cells were blocked for 30 minutes in a buffer containing 3% FBS and 1% BSA for one hour. Primary antibodies were prepared according to the instruction provided by the manufacturers and incubation was done for two to three hours on a shaker at normal temperature. After washing, the cells were incubated with secondary antibodies for 30 minutes. The fluorescent signals were acquired on a Zeiss Confocal Laser Scanning Microscope 710 (Carl Zeiss).

### Immunohistochemistry

All antibodies are listed in the previous section. Formalin-fixed paraffin-embedded (FFPE) sections of neoplastic human breast biopsies were deparaffinized by dipping the slides in xylene for 15 minutes. The sections were rehydrated by immersing them in serial dilution of ethanol for 5 minutes followed by rinsing. Antigen retrieval was performed by boiling the slides in citrate buffer (pH 6.0) for 15 minutes. Snap frozen sections of human xenograft tumors were thawed briefly, fixed and permeabilized as described above. Primary antibody incubation was carried out overnight at 4°C in a moisture chamber after a 30-minute blocking period. Secondary antibodies were incubated for one hour followed by several washes. Slides were then mounted with DAPI.

### shRNA transfection

Human shJagged1, scrambled lentiviral particles, and polybrene were purchased from Santa Cruz Biotechnology (USA). In summary, cells were cultured up to 50% confluence and were then treated with polybrene and lentiviral particles containing shRNA against Jagged1 or scrambled particles. Transfected cells were then selected using puromycin, and the down-regulation of Jagged1 was assessed by qPCR.

### RNA extraction & qPCR analysis

Total RNA was extracted with RNeasy Mini Kit (250) from Qiagen according to the manufacturer’s instructions. The RNA concentration was measured with Nanodrop 8000 spectrophotometer (Thermo Scientific) and 1 μg of RNA was used to produce cDNA with the ProtoScript M-MuLV *Taq* RT-PCR kit using the oligo dT primers (New England BioLabs). Semi-quantitative real-time analysis (qPCR) was done with a 7500 qPCR System (Applied Biosystems, USA) using a Go*Taq* 2-step RT-qPCR System (Promega) to amplify the gene of interest following the instructions provided. Primer sequences are listed in Additional file 1: Table S1.

### Protein extraction & western blot analysis

Cells were lysed in RIPA buffer (Sigma) containing protease and phosphatase inhibitors. For each sample, 40 μg of total protein were analyzed by Western blot. Proteins were separated on 10% SDS polyacrylamide gels and electroblotted at 4°C onto polyvinylidene difluoride (PVDF) membranes for one hour. The membranes were blocked in 5% nonfat dry milk or bovine serum albumin (BSA) in 0.1% Tween 20 in Tris-buffered saline prior to incubation with primary antibodies at 4°C overnight. The antibodies included phospho-Smad5 (1:500, Abcam, ab76296), phospho-Smad3 (1:500, Bioss, bs-3425R), Smad5 (1:1000, Cell Signaling, 9517), Smad3 (1:1000, Cell Signaling, 9523), Hes1 (1:200, Millipore), and β-actin (1:3000, Sigma, A2228). Blots were developed using HRP and chemiluminescence peroxidase substrate (ImmunoCruz) (Santa Cruz Biotechnology) and FluroChem HD2 (Cell Biosciences).

### RNA extraction & microarray analysis

RNA was isolated as explained above. Two quality control measures were carried out: (1) a spectrophotometric analysis and (2) a size fractionation procedure using a microfluidics instrument (Agilent Technologies). Total RNA (200 ng) was analyzed on Affymetrix GeneChip Human Genome U133 Plus 2.0 Array. Data were analyzed using Partek Software (V6.09.1110-6; Affimetrix), Venny online software (BioinfoGP; CNB-CSIC) and Ingenuity Pathway analysis (Ingenuity Systems, Redwood City, CA). Class comparisons between ECs^Norm^ and ECs^Mes^ (three biological replicates of each) were performed to identify gene expression changes with significant expression differences (*p* < 0.05) and two-fold increase or decrease in expression.

### Human xenograft tumor formation in NOD/SCID mice

All animal procedures were approved by the Ethics Committee for animal experimentation of Weill Cornell University (New York, USA). Six-week old female NOD/SCID mice were purchased from Jackson Laboratories. MDA-231 cells were injected (2 × 10^5^) with or without 2 × 10^6^ ECs (1:10 ratio) in the mice mammary fat pad of NOD/SCID mice. Four mice were assayed for each group. Each mouse received an injection of tumor cells on the left and co-injection of tumor and endothelial cells on the right side. The mice were euthanized and checked for tumor formation 18 and 30 days after inoculation. The extracted tumors were snap frozen for histological analysis.

### Ingenuity pathway analysis

We used Ingenuity Pathway Analysis software (IPA) (Ingenuity Systems, Redwood City, CA) for network analysis of EC genes that were differentially regulated upon co-culture. A global gene list was defined representing IPA keywords: “Metastasis”, “Proliferation of cell lines” and “Cell death of tumor cell line”. All edges are supported by at least one reference from the literature, textbooks, or canonical information stored in the Ingenuity Pathways knowledge database. *P-*values for enrichment of biological functions were generated based on hypergeometric distribution and calculated with the right-tailed Fisher's exact for 2 × 2 contingency tables as implemented in Ingenuity.

### Statistical analysis

All quantitative data are expressed as mean ± standard error of the mean (SEM). Statistical analysis and graphical presentation were performed using SigmaPlot 12 (Systat Software Inc., Chicago, IL) or Excel (Microsoft Corporation). A Shapiro-Wilk normality test, with a *p* = 0.05 rejection value, was used to test normal distribution of data prior to further analysis. All pairwise multiple comparisons were performed using one-way ANOVA followed by Holm-Sidak posthoc tests for data with normal distribution or, in case of a failed normality test, by Kruskal-Wallis analysis of variance on ranks followed by Tukey posthoc tests. Paired comparisons were performed using Student's t-tests or Mann–Whitney rank sum tests in case of unequal variance or failed normality test. Statistical significance was accepted for *p* < 0.05 (*), *p* < 0.01 (**) or *p* < 0.001 (***). All experiments were performed in triplicate and repeated three times (n = 3).

## Results

### Tumor-associated ECs enhance human xenograft tumor formation and demonstrate a mesenchymal phenotype in breast tumors

**Figure 1:**
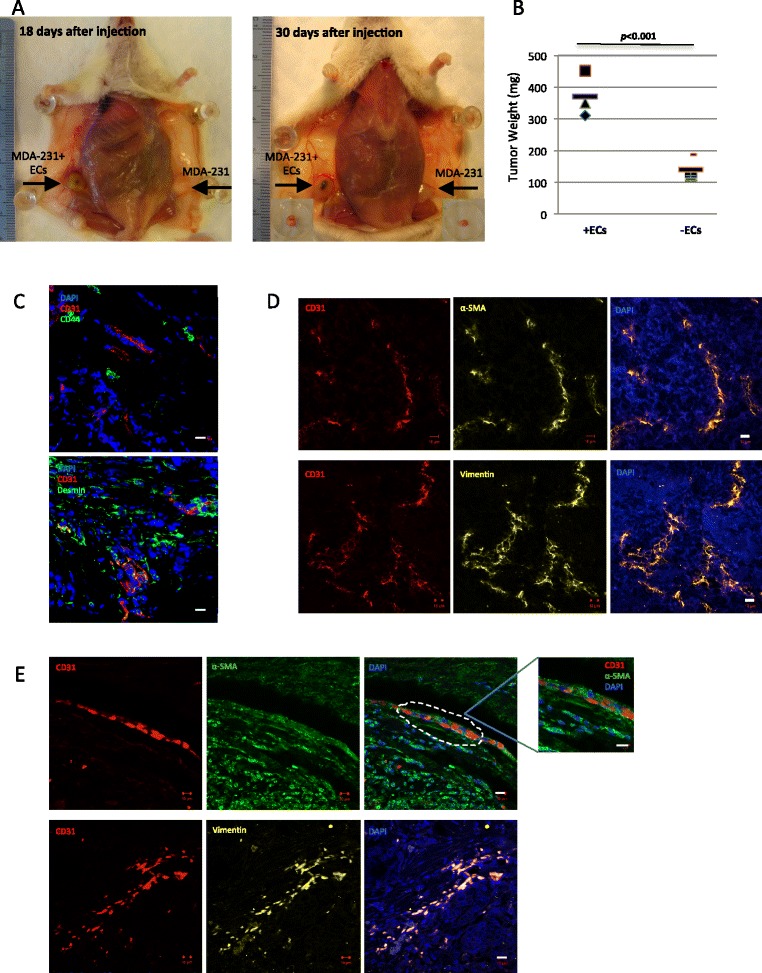
**Tumor-associated ECs enhance human xenograft tumor formation and demonstrate a mesenchymal phenotype in breast tumors. A)** NOD/SCID mice were sacrificed 18 and 30 days after subcutaneous injection with MDA-231 cells (2 × 10^5^ cells) with or without ECs (2 × 10^6^ cells) to make an assessment on xenograft tumor burden. Injection of MDA-231 cells without ECs (shown on the right side of each image) resulted in formation of non-angiogenic small tumors. In contrast, inoculation of MDA-231 cells mixed with ECs (1:10 ratio) (shown on the left side of each image) led to the generation of bulkier angiogenic tumors. **B)** Quantification of xenograft tumor weights formed in NOD/SCID mice injected by MDA-231 cells with and without ECs (***p < 0.001). **C)** Snap-frozen angiogenic xenograft tumors sections were stained with endothelial marker CD31 as well as vascular structural and functional markers CD44 and desmin to show the existence of viable capillaries in xenograft tumors. **D)** The xenograft tumor sections were stained with mesenchymal markers vimentin and α-SMA to demonstrate the acquisition of mesenchymal phenotype by tumor vasculature. **E)** Human neoplastic breast tissues were examined for the potential occurrence of mesenchymal properties in tumor endothelium *in vivo*. The tumor endothelium was first detected by CD31^+^ staining, and then examined for the existence of ECs showing mesenchymal phenotype within tumor by vimentin and α-SMA staining. Scale bars represent 10 μm.

To investigate the role of ECs in creating a pro-tumoral niche *in vivo*, we injected breast tumor cells MDA-231 to the mammary fat pad of NOD/SCID mice with or without ECs. The mice that were co-injected with MDA-231 and ECs showed significantly higher tumor burden with tumors weighing three-fold higher than mice that were only inoculated with MDA-231 cells (Figure 1A and B). Immunofluorescent imaging demonstrated that co-injected ECs generated functional and viable vessels in xenograft tumors (Figure 1C).

To confirm a mesenchymal phenotype in xenograft tumors, tumor sections were examined for the expression of mesenchymal markers such as vimentin and α-SMA. We showed co-expression of mesenchymal and endothelial markers in endothelium of the xenograft tumors (Figure 1D). In order to show that the tumor-stimulated mesenchymal properties in ECs might potentially occur *in vivo*, we stained FFPE sections obtained from breast tumor biopsies with mesenchymal markers. Similar to xenograft tumors, breast tumor endothelium showed a mesenchymal phenotype while maintaining an endothelial trait (Figure 1E).

### Breast cancer cells promote mesenchymal phenotypes in ECs

**Figure 2:**
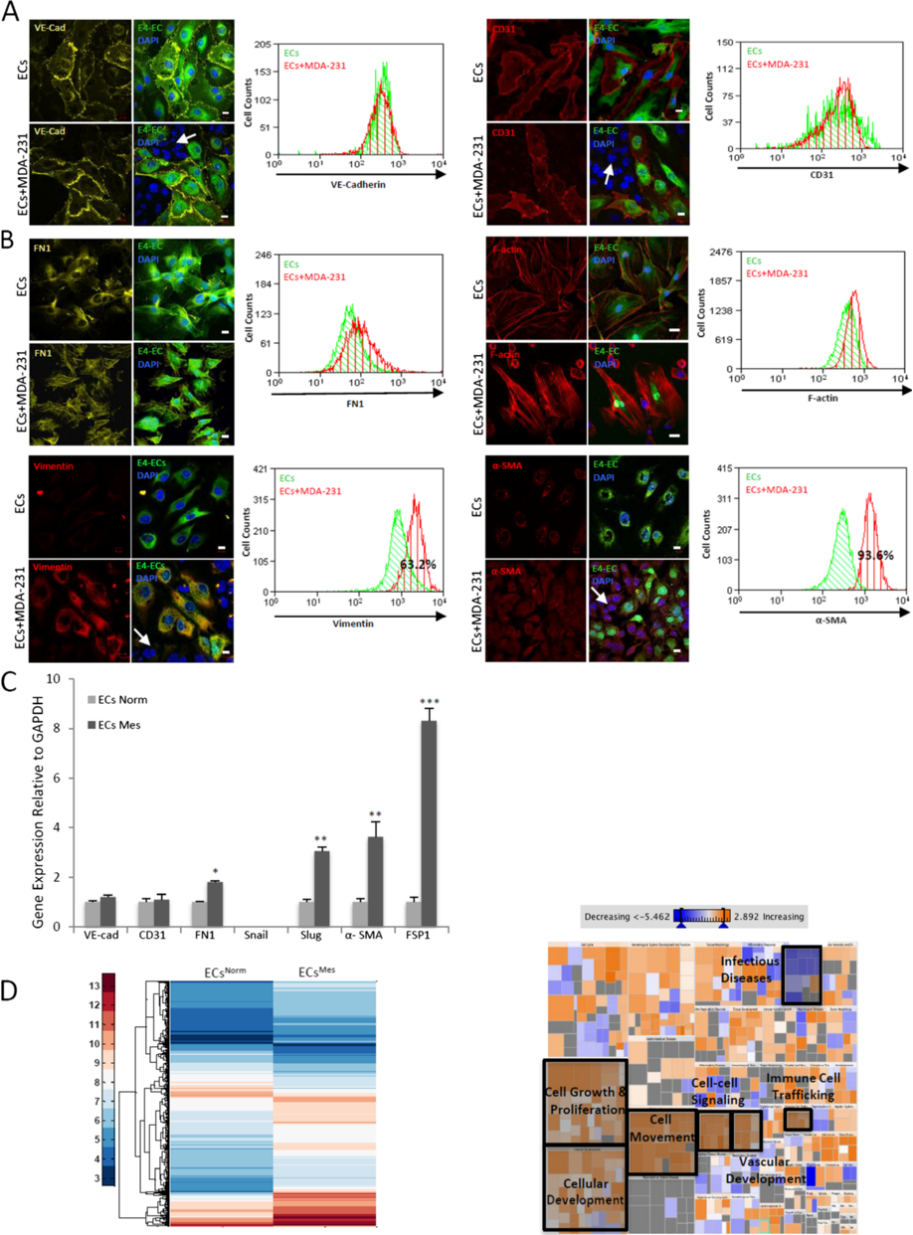
**Breast cancer cells promote mesenchymal phenotypes in ECs. A)** Confocal images and flow cytometry overlays showing the maintenance of endothelial phenotype in ECs grown in contact with MDA-231 BCCs (white arrows) as shown by CD31 (right panels) and VE-Cadherin (left panels) staining. **B)** Confocal images and flow cytometry overlays displaying the acquisition of mesenchymal phenotypes by ECs co-cultured in contact with MDA-231 BCCs (white arrows). GFP^+^ECs grown with MDA-231 cells show the appearance of mesenchymal phenotype by up-regulation of FN1, vimentin (left panels) α-SMA and F-actin stress fibers (right panels). Scale bars represent 10 μm. **C)** Semi-quantitative qPCR analysis further validated our results by showing that ECs^Mes^ maintained their endothelial phenotypes while over-expressed mesenchymal markers. (*** *p* < 0.001, **p < 0.01, *p < 0.05, mean ± SEM, n = 3). **D)** Heat map demonstrating differential gene expression in ECs^Mes^ (sorted from MDA-231cells) as compared to normal ECs (left). Functions treemap (right): rectangles represent ECs^Mes^ differential functions as compared to ECs^Norm^ based on literature data. Functions are grouped together into large clusters such as cellular growth and proliferation, cell cycle, cell movement etc. Rectangles are colored based on the z-score which is proportional to number of genes that are affected in any particular functional class with orange showing activation of that function and blue representing the inhibition of that function. In our dataset of ECs^Mes^, some functional groups showed consistent changes among all subgroups (shaded rectangles). These groups are cellular growth and proliferation (*p*-value 4.86E-29), cellular movement (*p*-Value 6.69E-19), cardiovascular system development (*p*-Value 8.30E-13), cell-to-cell signaling (*p*-value 8.74E-07), immune cell trafficking (*p*-Value 3.11E-06) and infectious diseases (*p*-Value 9.54E-14).

To show that cancer cells promote a mesenchymal phenotype in ECs, we initially used Human Umbilical Vein Endothelial Cells (HUVECs). Our flow cytometry results confirmed that MDA-231 cells stimulated a mesenchymal phenotype in HUVECs as shown by up-regulation of mesenchymal markers (α-SMA & FSP-1) while maintaining the endothelial trait (shown by CD31 & VE-Cadherin expression) (Additional file 1: Figure S1). Due to oversensitivity of HUVECs to starvation and rapid cell death, we used E4-ECs (ECs) that show relatively low *Akt* activation and are therefore resistant to serum-free conditions as well as tumor-cell induced apoptosis [19,21]. We further confirmed that cancer cells could stimulate a mesenchymal trait in ECs. The preservation of vascular phenotype in the co-culture setting was shown by stable expression of VE-cadherin and CD31 in ECs (Figure 2A). However, we were able to detect a shift toward mesenchymal phenotype represented by increased expressions of fibronectin (FN1), vimentin (Figure 2B, left panels), and α-SMA as well as stress fibers (F-actin) using confocal imaging (Figure 2B, right panels). These results were further validated by flow cytometry analyses (Figure 2A-B). Similar findings were obtained with ECs co-cultured with MCF-7 BCCs (Additional file 1: Figure S2A). Therefore, the ECs exposed to BCCs that show expression of mesenchymal markers are here referred to as “ECs^Mes^” and ECs with no exposure to BCCs are called “ECs^Norm^”.

Gene expression analysis by qPCR also demonstrated mesenchymal and endothelial phenotypes in ECs^Mes^ by increased expression of α-SMA and vimentin with stable expression of endothelial VE-Cadherin and CD31 markers (Figure 2C & Additional file 1: Figure S2B). We also checked if tumor-stimulated mesenchymal transition in ECs was contact-dependent by growing ECs in MDA-231 cells conditioned media (CM). We did not detect any changes in the expression of mesenchymal markers in ECs by flow cytometry (Additional file 1: Figure S3A-B).

Additionally, whole transcriptomics analysis was performed on ECs^Mes^ and ECs^Norm^. The results showed differential expression of over 1000 genes in ECs^Mes^ as presented in Figure 2D (left panel) (for a list of most significantly modified genes refer to Additional file 2: S4). IPA analysis revealed several functional pathways that were significantly up-regulated, and among these we identified several pathways compatible with a mesenchymal phenotypes such as cell growth and proliferation (*p*-Value 4.86E-29, 272 modified genes), cell movement (*p*-Value 6.69E-19, 164 modified genes), cell migration (*p*-Value 7.91E-19, 152 modified genes), vascular system development (*p*-Value 8.30E-13, 90 modified genes), blood vessel development (*p*-Value 3.97E-11, 72 modified genes), cell viability (*p*-Value 4.96E-15, 114 modified genes), and cell survival (*p*-Value 2.13E-16, 119 modified genes) (Figure 2D, right panel and Table 1).

**Table 1:** **Transcriptomics analysis of ECs** ^**Mes**^ **by Ingenuity Pathway Analysis (IPA) revealed differential expression of over one thousand genes compared to ECs** ^**Norm**^

**Gene name**	**Abbreviation**	**Fold change**	**Functional specification (IPA or GeneCards)**
**Cell crowth & proliferation**			
Epiregulin	EREG	+40.266	Cell proliferation and/or angiogenesis
Amphiregulin	AREG/AREGB	+19.911	Growth factor & mitogen
Baculoviral IAP	BIRC3	+6.765	Regulator of cell proliferation
Repeat containing 3			
Budding Un- inhibited			
By Benzimidazoles 1	BUB1	+6.160	Cell cycle regulator
NIMA-related kinase 2	NEK2	+5.968	Cell cycle regulator
Topoisomerase Iiα	TOP Iiα	+6.837	Regulator of mitosis
S100 calcium binding	PS100P	+6.466	Cell proliferation stimulator
Protein			
Vascular growth	VEGFC	+2.266	Endothelial cell proliferation
Factor C			
Human homolog of	MDM2	+2.515	Downstream effector of Akt signaling & enhancer of
Mouse double minute 2			endothelial cell survival
Baculoviral IAP Repeat	BIRC5	+3.308	Dual role in promoting proliferation & inhibiting apoptosis
Containing 5			
Insulin-like growth	IGFBP1	+13.116	Prolongs the half life of IGFs in cell growth promotion
Factor Binding Protein 1			
**Cell motility**			
S100 calcium binding	S100A4	+2.538	Cell motility & invasion
Protein A4			
Insulin-like growth	IGFBP1	+13.116	Cell migration
Factor Binding Protein 1			
Transforming growth	TGFBI	+2.934	Induced by TGFβ; has a role in inhibition of cell
Factor beta-induced			adhesion
Tetraspanin 8	TSPAN8	+2.461	Regulation of cell motility
Interleukin 6	IL6	+2.214	Induction of endothelial cell motility
Krupple-like Factor 5	KLF5	+3.685	Involvement in cell movement
Heparanase	HPSE	+2.501	Increase endothelial cell migration
Lectin galacroside-Binding	LGALS3	+2.397	Increase Endothelial cell migration
Soluble 3			
**Vascular development & angiogenesis**			
Jagged1	JAG1	+2.311	Cardiovascular development & angiogenesis
Interleukin 6	IL6	+2.214	Angiocrine factor
Vascular growth factor C	VEGFC	+2.266	Angiogenesis
Chemokine (C-X-C Motif)	CXCR4	+5.234	Regulator of vascular branching &
Receptor 4			endothelial processes remodeling
Heparanase	HPSE	+2.501	Increase endothelial cell migration
RUN-related Transcription	RUNX2	+6.952	Increase angiogenesis in endothelial cells
Factor 2			
Hairy/Enhancer of	HEY1	+2.644	possible role in vascular development
Split-related 1			

IPA functional clustering of the modified genes showed pathways activation compatible with a mesenchymal phenotype, including cell proliferation, cell motility and angiogenesis. Some genes in each functional category are shown in this table. For a complete list of genes, refer to Additional file 2: S4.

Altogether, our results showed that direct contact between BCCs and ECs promotes a mesenchymal shift in ECs^Mes^. While there is a mesenchymal transition in ECs^Mes^, their endothelial trait remains unchanged. This finding suggests that direct contact between ECs and tumor cells in specific areas of tumor microenvironment might create a distinct population of ECs with mesenchymal properties.

### Functional properties of ECs^Mes^

**Figure 3:**
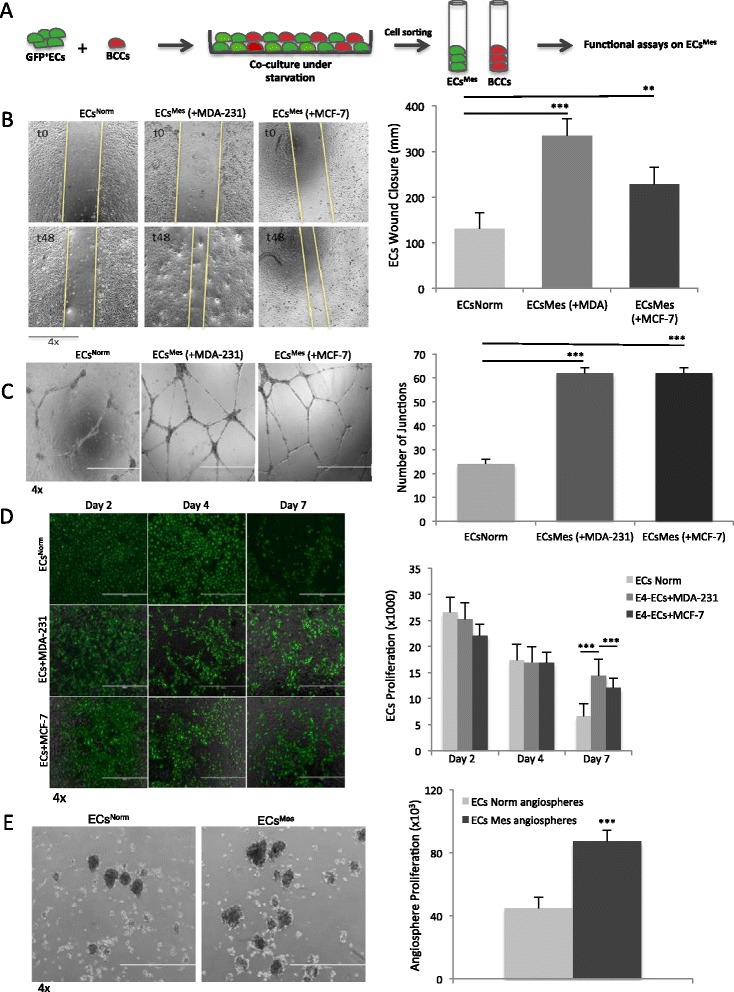
**Functional Properties of ECs** ^**Mes**^. **A)** Schematic representation of the experimental procedures for obtaining ECs^Mes^. BCCs were stained with a phycoerythrin (PE)-conjugated dye (PKH26) and co-cultured with GFP^+^ECs^Norm^ (1:10 ratio) for 3–5 days under starvation. Subsequently, GFP^+^ECs^Mes^ were sorted from PE^+^BCCs using GFP and PE fluorescence and ECs^Mes^ used for functional analyses. **B)** Wound healing assay to assess the migratory capacity of ECs^Mes^ as represented by their ability to close the wound (left panels). ECs^Mes^ were obtained by growing ECs^Norm^ with either MDA-231 (+MDA-231) or MCF-7 cells (+MCF-7). After sorting ECs^Mes^, a 48-hour wound healing assay was initiated under complete starvation to exclude the possibility of cell proliferation. ECs^Mes^ invasion property increased between 1.8 to 2.6-fold (right panel) compared to ECs^Norm^. **C)** Tube formation assay for comparing the angiogenic property of ECs^Mes^ with ECs^Norm^ (left panels). The assay was performed by growing ECs^Mes^ sorted from either MDA-231 (+MDA-231) or MCF-7 (+MCF-7) BCCs on matrigel overnight and estimating the number of capillary-like junctions. Quantitation of the junctions in ECs^Norm^ versus ECs^Mes^ demonstrated three-fold increase in angiogenic capacity of ECs^Mes^ (right panel). **D)** Cell proliferation assay and phase contrast imaging for evaluating survival capacity of ECs^Mes^. ECs^Norm^ were initially grown with MDA-231 or MCF-7 for five days to obtain ECs^Mes^. Then, BCCs and ECs^Mes^ were continued to grow together under starvation and their survival rate was compared to ECs^Norm^ grown without BCCs. Quantitative analysis estimated around two-fold increase in endurance capacity of ECs^Mes^ compared to ECs^Norm^ (right panel). **E)** An Angiosphere assay was used for evaluating the angiocrine property of ECs^Mes^. ECs^Norm^ or ECs^Mes^ were cultured under ultralow attachment condition to obtain angiospheres. A two-fold increase in angiosphere numbers was observed in ECs^Mes^ compared to ECs^Norm^. (*** *p* < 0.001, **p < 0.01, mean ± SEM, n = 3).

We hypothesized that the appearance of mesenchymal phenotype in ECs^Mes^ might instigate functional advantages. To test this hypothesis, we obtained ECs^Mes^ by co-cultivating ECs with MDA-231 or MCF-7 followed by sorting and subsequently evaluated their functional properties (Figure 3A & Additional file 1: Figure S5). Since an invasive endothelium was previously implicated in tumor development and metastasis [26], we compared the migration/invasion property of ECs^Mes^ with ECs^Norm^ by performing a wound healing assay. The results showed that ECs^Mes^ acquired an increased migration/invasion property (Figure 3B, left) as they closed the wound around three-fold faster than ECs^Norm^ (Figure 3B, right). It should be noted that the wound healing assay was performed over 48 hours and under complete starvation; therefore, wound closure was mainly the outcome of cell migration and the possibility of cell proliferation was excluded due to lack of serum and cytokines. To measure EC^Mes^ angiogenic potency in comparison to ECs^Norm^, a conventional tube forming assay was performed (Figure 3C, left). The results demonstrated three-fold increase in the capacity of ECs^Mes^ to form tubular branches on matrigel compared to their normal counterparts (Figure 3C, right). Since EC proliferation ensures angiogenesis and vasculogenesis [27,28], a proliferation assay was initiated to compare the self-renewal and survival capacity of ECs^Mes^ compared to ECs^Norm^. The results demonstrated significant improvement in survival but not proliferation rate of ECs^Mes^ (Figure 3D). Without BCCs, ECs^Norm^ survival rate was about two-fold lower on Day 7 (Figure 3D, right).

Finally, as has been recently shown by our group, EC activation triggers an angiocrine switch to allow secretion of angiocrine factors and cytokines that are primordial for tumor growth and tissue regeneration [7]. In our study, the sphere forming assay for constitution of angiospheres was used as a surrogate approach for enriching ECs with activated angiocrine phenotype. Concordantly, a colony forming assay was previously reported as one of several methods used to culture endothelial progenitor cells with neovascularization properties [29]. Hence, we benefitted from the sphere forming assay and cultured ECs^Mes^ under anchorage-independent condition for 3–5 days and compared their angiosphere (colony) forming capacity with ECs^Norm^. Consistent with our previous data, we showed that ECs^Mes^ displayed greater ability to form angiospheres as compared with ECs^Norm^ (Figure 3E). Collectively, our results confirmed a significant increase in cell plasticity toward acquisition of mesenchymal properties in ECs^Mes^ as shown by their enhanced migratory, angiogenic, angiocrine and survival properties under serum- and cytokine-free conditions.

To investigate the durability of the mesenchymal trait in ECs^Mes^ and to examine the importance of tumor context for its maintenance, we continued to grow and passage ECs^Mes^ for 10–15 days under normal conditions after sorting them from BCCs (Additional file 1: Figure S6A). ECs^Norm^ were cultured in parallel throughout the experiment to serve as controls. Interestingly, the mesenchymal phenotype was reversed in ECs^Mes^ 15 days after sorting from BCCs as indicated by down-regulation of mesenchymal markers by confocal imaging (Additional file 1: Figure S6B). Therefore, we refer to ECs with reversed mesenchymal phenotype as ECs^Reversed^. This observation was further confirmed at mRNA level by qPCR (Additional file 1: Figure S6C). To compare the functional properties of ECs^Reversed^ with the ECs^Mes^, we performed a wound healing assay that showed 1.5 to 2.4-fold decrease in ECs^Reversed^ ability to close the wound (Additional file 1: Figure S6D). Consistent with this observation, the result of our tube formation assay also demonstrated a 2.5-fold decrease in the number of tube junctions made by ECs^Reversed^ as compared to ECs^Mes^ (Additional file 1: Figure S6E).

### ECs^Mes^ provide a pro-tumoral niche for tumor growth and development

**Figure 4:**
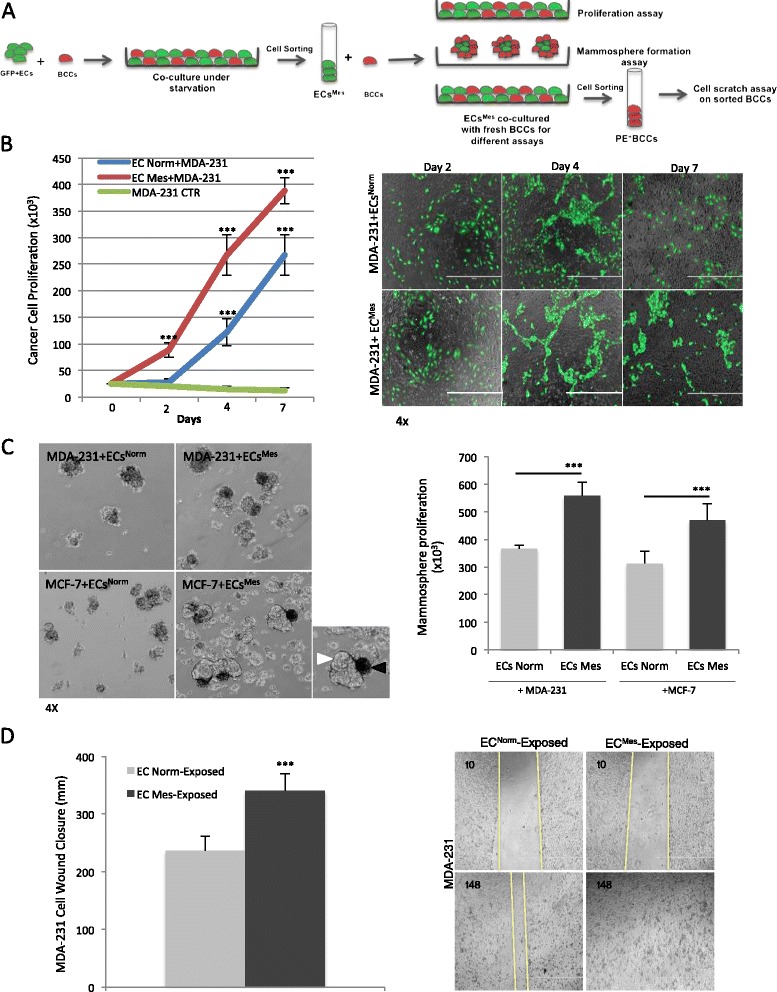
**ECs** ^**Mes**^ **provide a pro-tumoral niche for tumor growth and development. A)** Schematic representation of the experimental procedures carried out for evaluating the role of ECs^Mes^ in tumor development. ECs^Mes^ were acquired following the steps described in Figure 3A. Next, ECs^Mes^ and ECs^Norm^ were co-cultured with BCCs to compare their effects on cancer cell survival, cancer stem cell enrichment and cancer cell invasiveness. **B)** Cell proliferation assay and phase contrast imaging showing survival or proliferation of MDA-231 cancer cells when cultured alone or co-cultured with ECs^Mes^ or ECs^Norm^. Once MDA-231 cells were cultured alone under complete starvation, not only didn't they proliferate, they minimally survived the culture condition. However, once grown with ECs, they showed significantly higher proliferation rate. ECs^Mes^ were capable of increasing MDA-231 cell proliferation around 1.5-fold higher than ECs^Norm^. **C)** Mammosphere assay used for enriching cancer stem cells. MDA-231 cells were co-cultivated with either ECs^Norm^ or ECs^Mes^ under anchorage-independent condition for five days and their mammosphere-enriching capacity was evaluated by considering the number and size of the mammospheres grown in combination with ECs^Norm^ or ECs^Mes^. Angiospheres were excluded from quantification by their GFP signal. Around 1.6-fold increase was observed in the rate of mammosphere (white arrowhead) formation when they mingled with ECs^Mes^ (black arrowhead). **D)** Wound healing assay performed to assess the migratory capacity of breast tumor cells when grown and sorted from ECs^Mes^ or ECs^Norm^. BCCs exposed and sorted from ECs^Norm^ (ECs^Norm^-exposed) or ECs^Mes^ (ECs^Mes^-exposed) were used in a 48-hour wound healing assay under complete starvation to exclude the possibility of cell proliferation. BCCs that were grown and sorted from ECs^Mes^ were capable of closing the wound around 1.5-fold more efficiently than those sorted from EC^Norm^. (*** *p* < 0.001, mean ± SEM, n = 3).

To determine a role for ECs^Mes^ in breast cancer progression, we re-exposed ECs^Mes^ to freshly prepared BCCs and evaluated proliferation, sphere forming capacity and invasiveness of tumor cells grown with ECs^Mes^ or ECs^Norm^. The experimental procedure is illustrated in Figure 4A. Firstly, we compared BCC growth and survival under starvation in co-culture with ECs^Mes^ or ECs^Norm^ or alone. BCCs proliferation increased 1.5-fold, when grown with ECs^Mes^ compared to ECs^Norm^ (Figure 4B & Additional file 1: Figure S7). Notably, MDA-231 cells were capable of growth and proliferation once they were grown with ECs^Mes^ or ECs^Norm^ and once growing alone, they were unable to tolerate starvation and solely demonstrated minimal survival over 7 days. To see if the ECs role in proliferation or survival of tumor cells is contact-dependent, we cultured MDA-231 cells in serum/cytokine free CM of ECs^Norm^ or ECs^Mes^ for seven days. Our results did not show any significant increase in the survival rate of tumor cells grown in the CM of ECs^Norm^ or ECs^Mes^, which emphasize the importance of cell-to-cell contact between the two cell types for acquisition of functional advantages (Additional file 1: Figure S3C).

Then, we examined how ECs^Mes^ influence the enrichment of mammary stem cells (mammospheres). We co-cultivated GFP^+^ECs^Mes^ or GFP^+^ECs^Norm^ with BCCs under anchorage-independent conditions to expand mammary stem cells as “*mammo-angiospheres”* (Figure 4C, left panel). The results showed that ECs^Mes^ were capable of enhancing mammosphere growth about 1.6-fold compared to ECs^Norm^ (Figure 4C, right). We then investigated the effect of ECs^Mes^ on the invasiveness of BCCs. Therefore, BCCs were co-cultivated with either ECs^Mes^ or ECs^Norm^ and a wound healing assay was performed after sorting BCCs from the ECs. The results confirmed that ECs^Mes^ significantly enhance the migration capacity of MDA-231 cells in comparison with ECs^Norm^ (Figure 4D).

Our results indicate that cancer–endothelial crosstalk potentially modulates EC plasticity; contact with tumor cells triggers a mesenchymal (activated) phenotype in ECs that is reversed once the tumoral context is removed. Thus, cell-to-cell contact seems to be crucial for the initiation and maintenance of the mesenchymal state of ECs^Mes^ and may be regarded as a novel approach for treating cancer. However, a better understanding of the molecular mechanism regulating this interaction seems necessary.

### Tumor-stimulated mesenchymal phenotypes in ECs^Mes^ is regulated by synergistic Notch and TGFβ pathways

**Figure 5:**
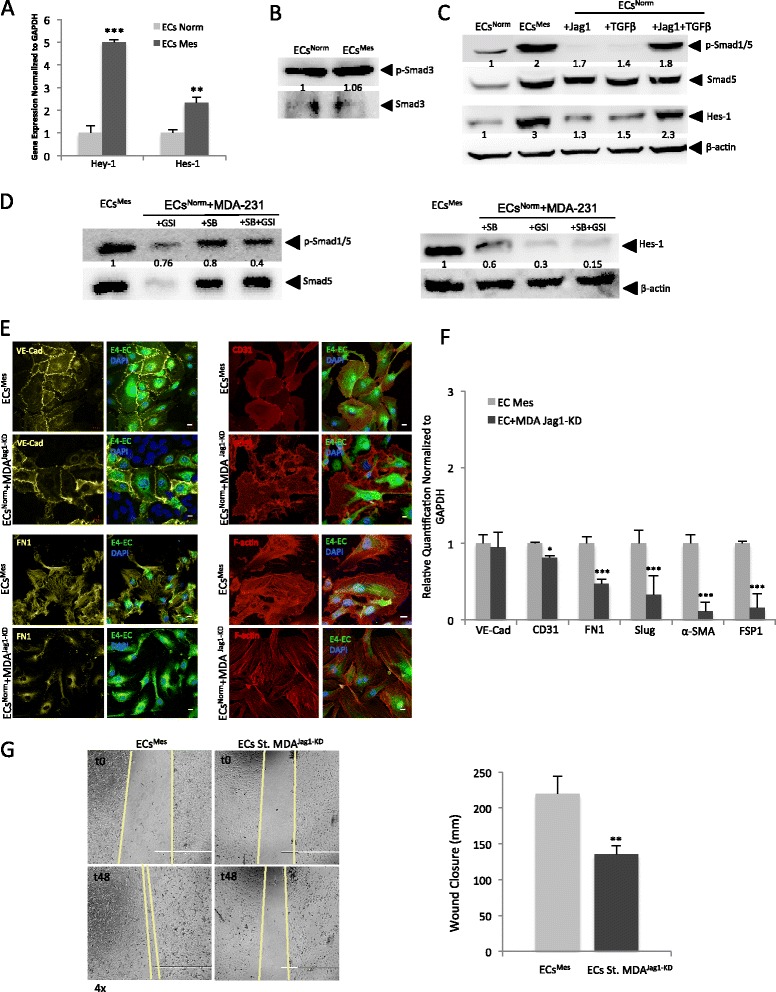
**Mesenchymal phenotypes in ECs** ^**Mes**^ **is regulated by synergistic Notch and TGFβ pathways. A)** Semi-quantitative qPCR showing the over-expression of notch transcription factors in sorted ECs^Mes^. (*** *p* < 0.001, ** *p* < 0.01, mean ± SEM, n = 3). **B)** Western analysis showing stable p-Smad3 expression in ECs^Norm^ and ECs^Mes^ compared to total protein. **C)** Western analysis demonstrating the activation of Smad5 in ECs^Mes^ compared to ECs^Norm^ (top lane). Once ECs^Norm^ were treated with Jag1 and/or TGFβ1, p-Smad5 was up-regulated (top lane). Hes-1 up-regulation in response to TGFβ1 or Jag1 treatments suggests notch pathway involvement (third lane). Simultaneous supplementation of ligands triggered dramatic Hes-1 up-regulation in ECs^Norm^ implying a synergistic effect. **D)** Western analysis displaying p-Smad1/5 down-regulation in response to notch and/or TGFβ inhibition. GSI and SB were added to ECs^Norm^-MDA-231 co-cultures, then ECs were sorted for analyzing p-Smad1/5 down-regulation comparing to total protein (left panel). Inhibition also down-regulated Hes-1 activation (right panel). Concurrent inhibitions of ECs^Mes^ dramatically reduced p-Smad1/5 and Hes-1 activation. **E)** Confocal images demonstrate endothelial and mesenchymal states of ECs co-cultured with MDA-231^Jag1-KD^ or MDA-231Scrambled. ECs grown with MDA-231^Jag1-KD^ demonstrated normal endothelial feature (Upper panels). However, they displayed decreased mesenchymal phenotype compared to ECs^Mes^ (sorted from MdA-231Scrambled) (bottom panel). Scales are 10 μm. **F)** Semi-quantitative qPCR compared expression of endothelial and mesenchymal markers in ECs^Mes^ and ECs sorted from MDA-231^Jag1-KD^ cells. Silencing Jag1 and notch down-regulation in MDA-231^Jag1-KD^ counteracted the acquisition of mesenchymal phenotype by ECs. **G)** Wound healing assay to assess the migratory capacity of ECs sorted from MDA-231^Jag1-KD^ (ECs St. MDA-231^Jag1-KD^) and ECMes (sorted from MDA-231^Scrambled^) (left panels). After sorting, a 48-hour wound healing assay was initiated under complete starvation to exclude the possibility of cell proliferation. The invasion property of ECs St. MDA-231^Jag1-KD^ decreased by 1.6-fold compared to ECs^Mes^ (right panel).

Previous works demonstrated that TGFβ or notch pathways regulate the EndMT phenomenon during normal and pathological developmental processes [17,18,30-33]. To investigate the involvement of notch or TGFβ pathways in tumor-fostered mesenchymal shift in ECs^Mes^, we first examined whether notch and/or TGFβ downstream effector molecules are activated in ECs^Mes^ compared to ECs^Norm^. Our qPCR results demonstrated the up-regulation of notch downstream transcription factors Hey-1 and Hes-1 in ECs^Mes^ (Figure 5A). Concordantly, our transcriptomics results confirmed the up-regulation of several genes involved in EMT or EndMT processes in ECs^Mes^, among which a notch receptor as well as signaling effectors like Hey-1 and Hes-4 were listed (Table 2).

**Table 2:** **Transcriptomics analysis by IPA showed the up-regulation of several genes in ECs** ^**Mes**^ **that are involved in EMT/EndMT processes some of which are listed in the table**

**Gene name**	**Function**	**Fold change**	**Refs**
**S100A4 (FSP1)**	Mesenchymal marker	+2.54	[1,17,18]
**Jagged1**	Notch signaling ligand	+2.31	[32-34]
**Notch2**	Jagged1 receptor	+2.12	[32-34]
**HEY1**	Notch effector	+2.64	[62,63]
**HES4**	Notch effector	+2.1	[62,63]
**TGFBI**	Involvement in EMT	+2.93	[18,30,31,34]
**BMPR1A**	TGFβ/Smad receptor	+2.80	[34,35]
**EGFR**	EMT inducer	+3.78	[64,65]
**WNT5B**	EMT inducer	+2.23	[66-68]
**HMGA2**	EMT-inducing transcribtion factor	+2.13	[69-71]
**IGFBP2**	EMT-inducing signaling	+13.12	[72,73]
**STAT2**	EMT-inducing signaling	+3.1	[72,74]

Earlier studies pointed out the involvement of distinct cellular receptors, TGFβ/ALK1 (BMPs) triggering Smad1/5/8 phosphorylation and TGFβ/ALK5 (activin) stimulating Smad2/3 phosphorylation in endothelial transformation [34]. Our western analysis ruled out the activation of smad3 signaling in ECs^Mes^ (Figure 5B). However, our results determined the activation of Smad5 in ECs^Mes^ (Figure 5C, top lane). Also, transcriptomics data confirmed the up-regulation of BMPR1A receptor in ECs^Mes^, which was previously shown to mediate BMP-2 and BMP-4 effect in Smad signaling activation (Table 2) [34,35].

To confirm a role for endothelial notch and TGFβ in phosphorylation of Smad5 in ECs^Mes^, ECs^Norm^ were treated with soluble forms of human recombinant Jag1 (4 μg/mL) and TGFβ1 ligands (5 ng/mL). Our western analysis showed Smad5 phosphorylation only in response to treating ECs^Norm^ with Jag1 and TGFβ ligands as was compared with total Smad5 protein (Figure 5C, top lane). To further verify the synergistic role of Jag1/notch and TGFβ/Smad5 in this process, we treated ECs^Norm^ with both Jag1 and TGFβ ligands and observed increased level of Smad5 phosphorylation (Figure 5C, top lane) confirming the synergistic role for the ligands in activation of Smad5. To further validate a role for notch pathway in this process, we showed up-regulation of notch pathway downstream effector Hes-1 in response to treating ECs^Norm^ with Jag1 and/or TGFβ ligands (Figure 5C, third lane). Band densitometry analyses showed the highest level of Smad5 and Hes-1 activation once ECs^Norm^ were treated with both ligands simultaneously (Additional file 1: Figure S8-A).

Additionally, we treated co-cultures of ECs^Norm^ and MDA-231 cells with inhibitors of notch (GSI, 5 μM) and TGFβ (SB-431542, 10 μM) pathways. After sorting ECs, phosphorylation of Smad5 was compared with ECs^Mes^ by western blotting. The results demonstrated that notch and TGFβ inhibition reduced Smad5 phosphorylation (Figure 5D, left panel). Additionally, we showed that inhibition of notch and TGFβ pathways down-regulated Hes-1 protein (Figure 5D, right panel). Band densitometry analysis confirmed the highest p-Smad5 and Hes-1 inhibition once ECs^Mes^ were concurrently treated with the inhibitors (Additional file 1: Figure S8B). Besides, our results indicated that mesenchymal phenotype in ECs^Mes^ were down-regulated when they were treated with GSI and SB (Additional file 1: Figure S8C). Collectively, our findings suggest a synergistic role for endothelial Jag1 and TGFβ in regulating tumor-stimulated mesenchymal phenotypes in ECs^Mes^.

To further validate our results, we established a stable population of MDA-231^Jag1-KD^ by using shRNA against Jagged1 on tumor cells and determined Jagged1 down-regulation compared to MDA-231^Scrambled^ by qPCR (Additional file 1: Figure S8D). Then, down-regulation of notch downstream effectors such as Hey-1 and Hes-1 was compared between ECs that were exposed and sorted from MDA-231^Scrambled^ or MDA-231^Jag1-KD^ (Additional file 1: Figure S8E). Next, the endothelial and mesenchymal phenotype of ECs co-cultured with MDA-231^Jag1-KD^ was compared to ECs^Mes^ that were grown with MDA-231^Scrambled^. The confocal results confirmed stable expression of endothelial and mesenchymal markers such as VE-Cadherin, CD31 in ECs grown with MDA-231^Jag1-KD^ (Figure 5E, top). However, mesenchymal markers such as FN1 and F-actin were not up-regulated in those cells (Figure 5E, bottom). These results were further validated by qPCR analysis (Figure 5F). Moreover, in order to evaluate the migratory potential of ECs sorted from MDA-231^Jag1-KD^ (ECs St. MDA^Jag1-KD^) compared to ECs^Mes^ (ECs St. MDA-231^Scrambled^), a wound-healing assay was performed. We observed a reduction in ECs ability to close the gap when grown and sorted from MDA-231^Jag1-KD^ (Figure 5G). Overall, these findings confirm the involvement of notch pathway in regulation of tumor-promoted mesenchymal transitions in ECs^Mes^.

## Discussion

Our main finding is that the tumor cells promote the acquisition of a transient contact-dependent mesenchymal phenotype in ECs contributing to the generation of a pro-tumoral niche. Transforming growth factorβ (TGFβ) and notch pathways seem to be determinant inducers of tumor-fostered mesenchymal phenotype in ECs.

The tumor microenvironment is implicated in the propagation and metastasis of several tumor types [36-38]. The role of endothelial cells (ECs) -as one the components of tumor stroma- in cancer development were merely thought to involve angiogenesis [26]. Recently, our team demonstrated a novel role for tumor endothelium in tissue repair, self-renewal of HSCs as well as tumor growth and stemness by *angiocrine factors* [7,8,11,13]. In addition, ECs were previously described as cells that demonstrated a high degree of plasticity under different conditions, a feature that is implicated in tumor development [1,6]. Tumor-associated endothelial plasticity may be explained in the context of spatiotemporal plasticity (i.e., to change phenotype and function) and reciprocity (i.e., by processing signals received from the environment) that have been earlier explained by Bissell’s group to be fundamental in step-wise changes in both tumor cells and their microenvironment [39-41]. Accordingly, plasticity and reciprocity account for the morphologic and functional heterogeneity driven by mechanisms such as cell-to-cell signaling to allow cells to cope with altering environmental conditions [42-44]. These mechanisms might be modulated by tumor and stromal cells crosstalk to co-evolve in a dynamic microenvironment [44,45]. Hence, our work emphasizes a crosstalk mechanism that is absolutely dependent on cell-to-cell contact between tumor and endothelium. The focal points of interaction between tumor and vasculature might not be abundantly present within the tumor bulk, but may potentially serve as miniature sites within the tumor microenvironment that may enhance neovascularization leading to increased tumor growth and metastasis.

In this study, we demonstrated that tumor cells are capable of promoting mesenchymal phenotype in their neighboring endothelium. In return, the ECs^Mes^ significantly contributed to tumor development. Although we initially observed this phenomenon in HUVECs, we continued our work with the widely used E4-ECs [21] to circumvent the hurdle of tumor-induced HUVEC apoptosis in co-cultures that was also reported previously by Kebers et al. [19]. The mesenchymal transformation in tumor endothelium in conjunction with loss of endothelial phenotype has been previously described in EndMT phenomenon as a mechanism for generation of CAFs [17,18]. However, the acquisition of mesenchymal phenotype with maintenance of endothelial trait and its significance for tumor propagation has never been explained earlier. The importance of this phenomenon may be explained by enhanced survival, mobility and angiocrine properties as well as the angiogenic ability of ECs^Mes^. Our transcriptomics data further validate our hypothesis by showing up-regulation of pathways involved in cell development, signaling, and movement in addition to vascular system expansion and blood vessel formation.

The present work also focused on looking into the role of mesenchymal endothelium (ECs^Mes^) in breast tumor progression. *In vivo*, human xenograft tumor formation was enhanced by co-injection of ECs and tumor cells in NOD/SCID mice showing the up-regulation of mesenchymal markers in tumor-associated ECs. Also, by developing adherent (2D) and non-adherent (3D) co-culture systems, the role of ECs^Mes^ in cancer proliferation, stemness and invasiveness was evaluated. Based on a study by Maffini et al., the preliminary target for a carcinogen is tumor stroma and mutations in mammary epithelial cells are not sufficient for tumor initiation [46]. Campbell’s group earlier demonstrated that a modified stroma preferentially promotes the outgrowth of abnormal epithelial cells [44,47]. Also, a study conducted by Moses and colleagues demonstrated that cell signaling abnormalities in stromal fibroblasts promoted mammary tumorigenesis in a non-cell-autonomous manner [48,49]. Therefore, it is primordial to study tumor microenvironmental changes that occur during cancer progression. In addition to influencing tumor initiation and progression, these changes significantly impact the efficacy of cancer therapy specifically when targeting stroma-regulated pathways [50]. In accordance with these reports, our work highlighted the importance of tumor contexture in fostering phenotypic changes in ECs^Mes^ and how this alteration impact tumor proliferation, survival, stemness and pro-metastatic properties. Therefore, elucidation of the mechanisms underlying microenvironment alteration will be beneficial in targeting stroma to combat cancer.

TGFβ was previously suggested as an important role player in the EndMT process during normal and pathological situations [30,51,52]. In addition, notch signaling was shown to promote EndMT during both cardiac development and oncogenic transformation [32,33]. In this study, we showed a similar phenomenon through which tumor cells enhanced mesenchymal phenotypes in ECs while preserving the endothelial phenotypes. We showed that the tumor-stimulated processes leading to creation of EC^Mes^ are also mediated by phosphorylation of TGFβ/Smad1/5 in synergy with notch pathway activation. Notch involvement in the regulation of TGFβ signaling in ECs was previously reported [32,34,53,54]. Both synergy and antagonism between notch and TGFβ signaling were described in ECs in a context-dependent manner [34,53-59]. Our data confirmed that tumor-activated TGFβ/Smad1/5 phosphorylation was regulated by synergistic activation of notch and TGFβ pathway by showing that TGFβ and Jag1 were capable of inducing Smad5 phosphorylation as well as Hes1 up-regulation. Simultaneous inhibition of notch and TGFβ pathways not only impaired Smad5 phosphorylation but also impaired the acquisition of mesenchymal traits by ECs.

Earlier reports by Karsan’s group emphasized the importance of Smad1/5 phosphorylation in promoting proliferation and migration of ECs [34]. Also, phosphorylation of Smad1/5/8 was implicated in ECs migration [60]. Consistent with these observations, we demonstrated that tumor interaction with ECs stimulated TGFβ/Smad1/5 phosphorylation possibly resulting in gain of functional advantages by ECs^Mes^. Since the acquired mesenchymal phenotypes in ECs^Mes^ seem to be a reversible phenomenon, targeted inhibition of the molecular modulators of this process may therefore be considered as potential therapeutic approaches.

Our findings of the tumor-promoted mesenchymal phenotype in ECs^Mes^ might partly explain the molecular mechanisms that govern ECs plasticity. Interestingly, our transcriptomics data while confirming mesenchymal traits, demonstrated modifications of over 1000 genes that might be relevant to tumor endothelial biology and play roles in phenomena such as resistance to treatment and metastasis. As such, up-regulation of previously described angiocrine factors such as IL6, Jag1, and CXCR4 [61] in ECs^Mes^ may have potentially participated in the constitution of a pro-tumoral niche; however, the exact determinants of the angiocrine niche need to be clarified in future studies. While in our settings a change to ECs^Mes^ found to be transient, its permanence has not yet been carefully addressed in any study. However, the constitution of a transient niche might offer a window for the constitution of residual or resistant disease. Additional investigations involving *in vivo* approach are required to validate our data in order to design new drugs for impairing tumor-EC interaction as a mean to treat cancer.

## Conclusion

Although the importance of microenvironment modification in tumor development is already known, the molecular changes underlying these alterations is not well recognized. ECs are essential components of microenvironment, which influence tumor progression by establishing the tumor vascular niche and producing angiocrine factors. In addition, ECs seem to adapt to the context they reside in by showing plasticity. This study demonstrated that in tumor microenvironment, ECs are instructed through tumor-derived inducers to proceed to an activation state characterized by up-regulation of mesenchymal phenotypes. Since this interaction strongly relies on cell-to-cell contact between tumor cell and endothelium, it may not be widely detectable in tumor microenvironment. Where the cell interaction occurs, the new mesenchymal trait along with the intrinsic endothelial properties may allow ECs^Mes^ to create a pro-tumoral niche supporting tumor progression and metastasis. Since EC^Mes^ mesenchymal characteristic is transient and strongly dependent on tumor contexture, it may introduce a new therapeutic target for treating cancer.

## Additional files

Additional file 1: Figure S1. Mesenchymal phenotype is simulated in HUVECs through contact with tumor cells. **Figure S2.** Mesenchymal phenotype is initiated in ECs through contact with MCF-7. **Figure S3.** MDA-231 conditioned media (CM) does not induce a mesenchymal phenotype in ECs. **Figure S5.** Flow cytometry scatter plots illustrate gating for PE+MDA-231 and GFP+ECs for sorting. **Figure S6.** Mesenchymal phenotype is reversed once EC contact with cancer cells is disrupted. Figure S7. Proliferation of MCF 7 in co culture with ECs^Norm^ or ECs^Mes^. **Figure S8. Table S1.** List of Primers. Additional file 2:
**S4.** An Excel sheet containing full gene list of the IPA functional gene clustering has been uploaded for creation of a hyperlink.

## Abbreviations

BCCs: Breast cancer cells
BCSCs: Breast cancer stem cells
BMP: Bone morphogenic protein
CAFs: Cancer-associated fibroblasts
CM: Conditioned media
ECs (E4-ECs): Endothelial cells transfected with *E4ORF1* gene
ECs^Norm^: Endothelial cells with no contact with BCCs
ECs^Mes^: Endothelial cells exposed to BCCs and showing mesenchymal phenotype
ECs^Reversed^: Endothelial cells with reversed mesenchymal phenotype
EMT: Epithelial-to-mesenchymal transition
EndMT: Endothelial-to-mesenchymal transition
E4orf1: Early 4 region open reading frame 1
FFPE: Formalin-fixed paraffin-embedded
FN1: Fibronectin 1
FSP-1: Fibroblast specific protein
GSI: γ-secretase inhibitor
HUVECs: Human umbilical vein endothelial cells
Jag1: Notch pathway ligand
MDA-231^Jag1-KD^: MDA-231 cells with Jagged1 knock-down
α-SMA: Alpha smooth muscle actin
TGFβ1: Transforming growth factor β1
