# Supplementary material for: Breast cancer cells promote a notch-dependent mesenchymal phenotype in endothelial cells participating to a pro-tumoral niche
Source: J Transl Med. 2015 Jan 27;13:27. doi: 10.1186/s12967-015-0386-3 (PMC4336716; doi:10.1186/s12967-015-0386-3)
Supplement: Additional file 1: Figure S1. — Mesenchymal phenotype is simulated in HUVECs through contact with tumor cells. Figure S2. Mesenchymal phenotype is initiated in ECs through contact with MCF-7. Figure S3. MDA-231 conditioned media (CM) does not induce a mesenchymal phenotype in ECs. Figure S5. Flow cytometry scatter plots illustrate gating for PE+MDA-231 and GFP+ECs for sorting. Figure S6. Mesenchymal phenotype is reversed once EC contact with cancer cells is disrupted. Figure S7. Proliferation of MCF 7 in co culture with ECsNorm or ECsMes. Figure S8. Table S1. List of Primers. [file 12967_2015_386_MOESM1_ESM.pdf]

Supplementary Figures & Legends

Figure S1. Mesenchymal phenotype is induced in HUVECs through contact with tumor cells

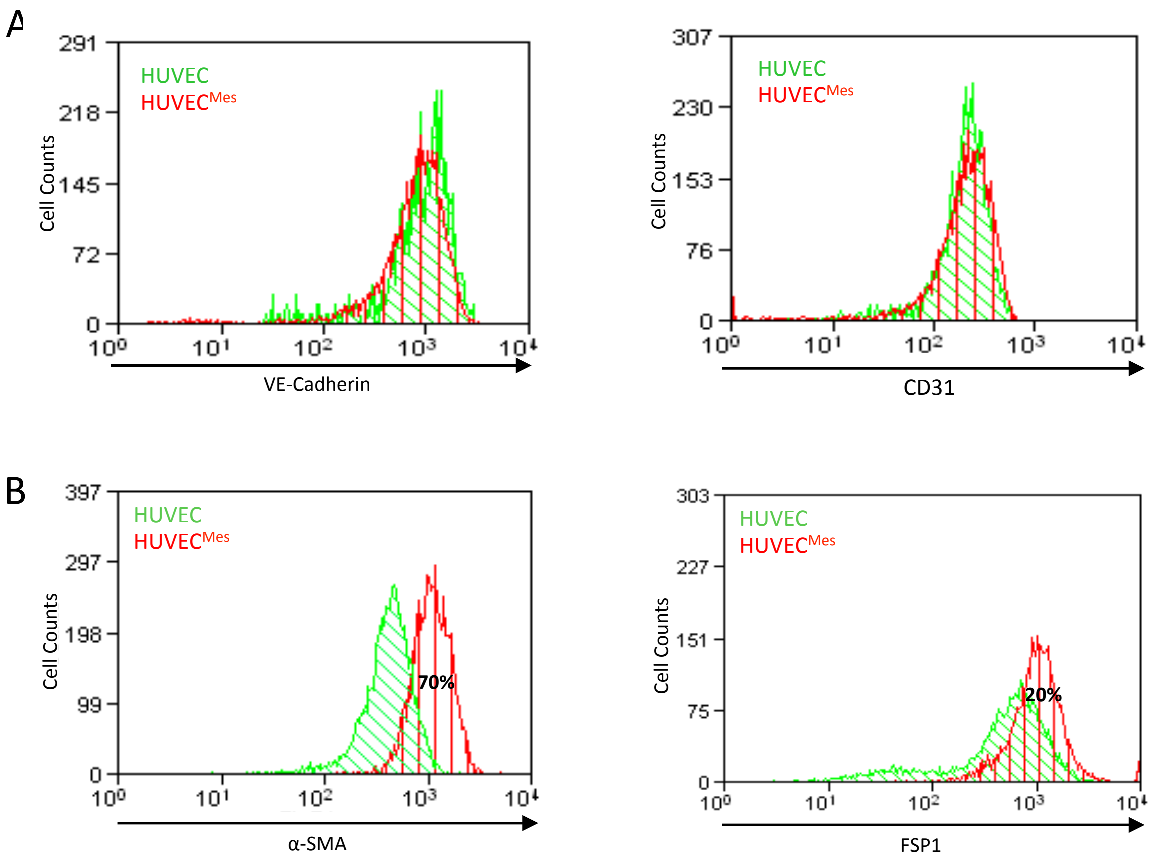

A) Flow cytometry overlays demonstrating stable expression of endothelial markers CD31 and VE-Cadherin in HUVECs after having contact with MDA-231 tumor cells. HUVECs were co-cultivated with MDA-231 breast cancer cells under starvation for 3-5 days, stained with endothelial markers (CD31 and VE-Cadherin) and analyzed by flow cytometry.

B) Flow cytometry overlays displaying the up-regulation of mesenchymal phenotypes in HUVECs after contact with MDA-231 BCCs. Our results confirmed the acquisition of mesenchymal phenotype by HUVECs by showing the up-regulation of α-SMA (about 70%) and FSP-1 (about 20%) of of HUVECs.

Figure S2. Mesenchymal phenotype is initiated in ECs through contact with MCF-7

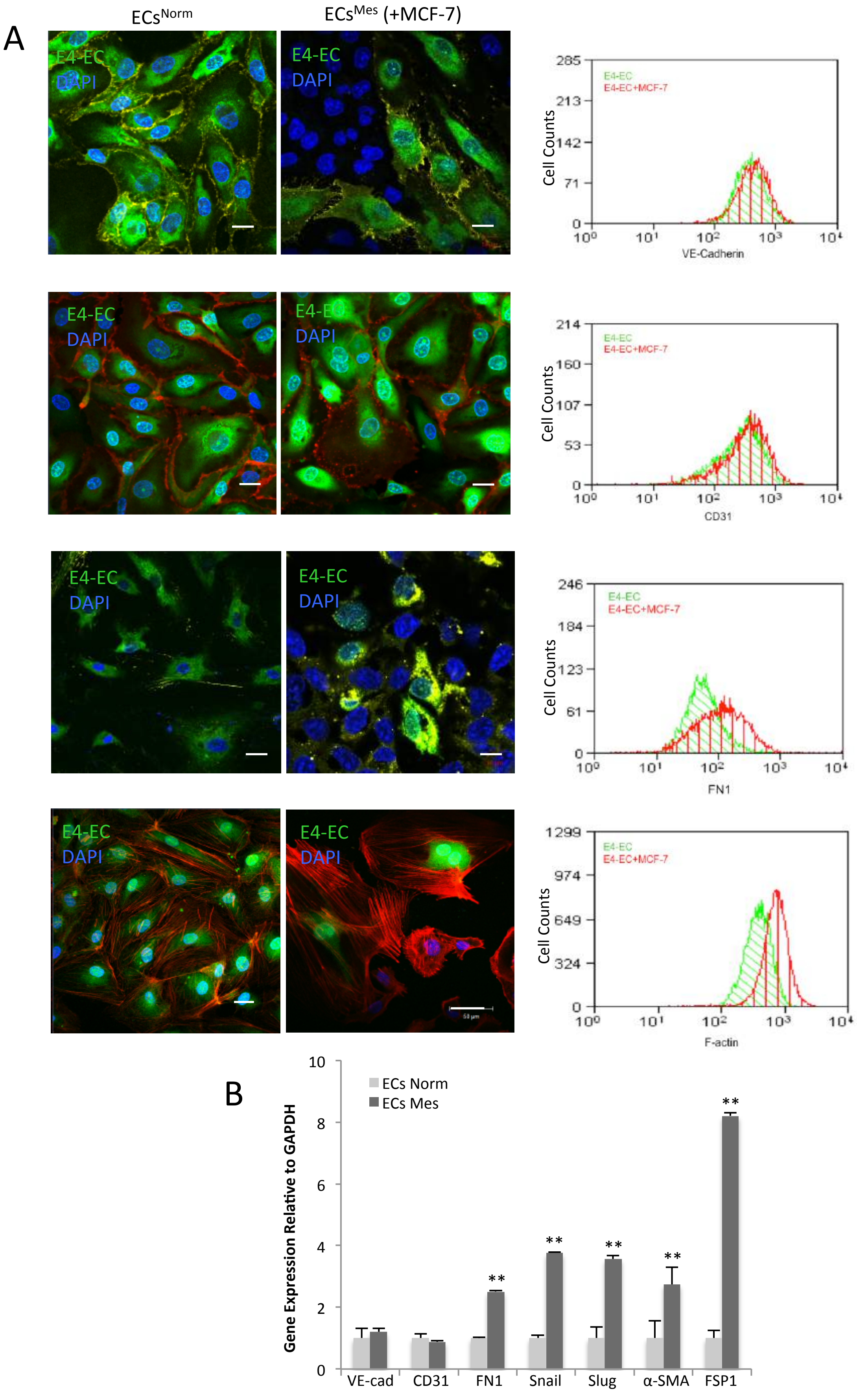

Figure S2. Mesenchymal phenotype is initiated in ECs through contact with MCF-7 breast cancer cells

A) Confocal images and flow cytometry overlays show that endothelial phenotype in ECs^{Mes} is maintained after contact with MCF-7 BCCs as shown by CD31 and VE-Cadherin staining. However, our results confirmed that MCF-7 cells were capable of inducing mesenchymal phenotype in ECs^{Mes} as demonstrated by up-regulation of FN1, vimentin (left panels) α-SMA and F-actin stress fibers (right panels). White arrows in some images show the MCF-7 cells in co-culture with GFP^{+}ECs. Scale bars represent 20 µm.

B) Semi-quantitative qPCR further validated our results by showing that ECs^{Mes} sorted from MCF-7 BCCs also maintained their endothelial phenotypes while over-expressed mesenchymal markers. (**p<0.01, mean ±SEM, n=3)

Figure S3. MDA-231 conditioned media (CM) does not induce a mesenchymal phenotype in ECs.

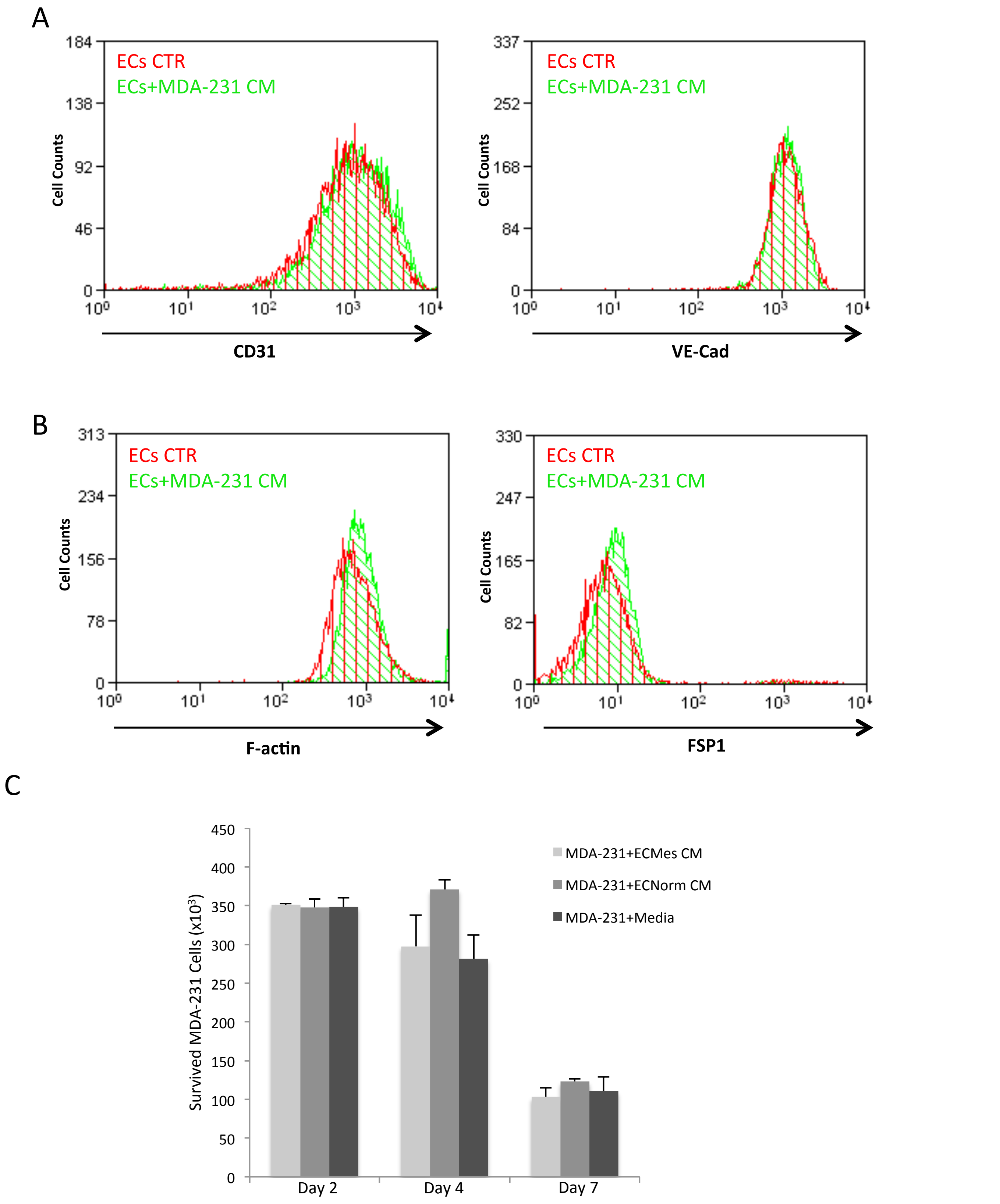

Figure S3. MDA-231 conditioned media (CM) does not induce a mesenchymal phenotype in ECs.

A) Flow cytometry overlays demonstrating stable expression of endothelial markers CD31 and VE-Cadherin in ECs grown in MDA-231 starvation CM for 3-5 days.

B) Flow cytometry overlays displaying stable mesenchymal phenotypes in ECs grown in MDA-231 starvation CM. Our results confirmed the importance of direct cell-to-cell contact between BCCs and ECs in triggering the transition to a mesenchymal phenotype in ECs.

S4. An Excel sheet containing full gene list of the IPA functional gene clustering has been uploaded for creation of a hyperlink.

Figure S5. Flow cytometry scatter plots illustrate gating for PE+MDA-231 and GFP+ECs for sorting

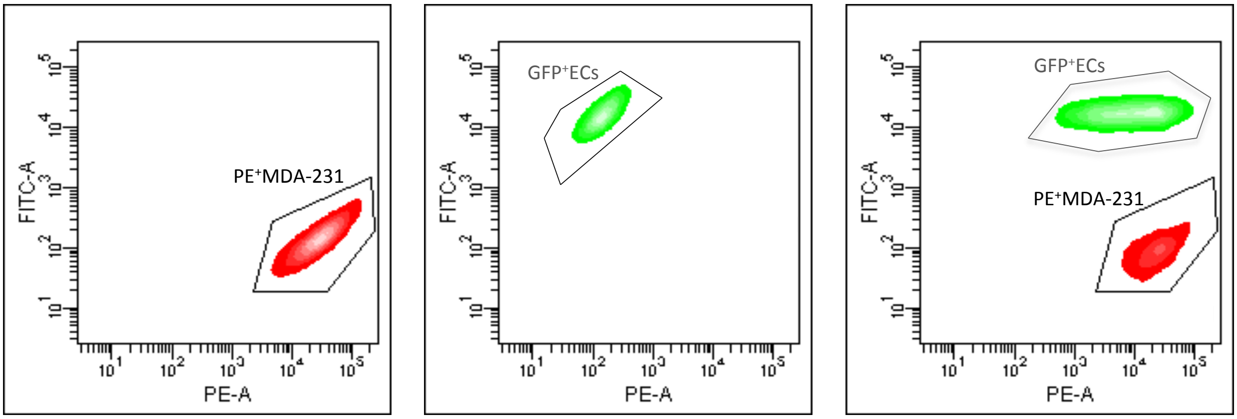

BCCs were stained with a PE-conjugated PKH26 dye (Sigma) before a co-culture was set up with GFP +ECs. After 3-5 days of co-cultivation, BCCs and ECs were gated using their PE and GFP fluorescent, respectively. GFP fluorescence was acquired using 488 nm blue laser and 510/50 nm emission. PE fluorescent was acquired using 496/566 nm blue laser and 576 nm emission to separate BCCs from GFP +ECs and sorting was done using purity masks.

Figure S6. Mesenchymal phenotype is reversed once EC contact with cancer cells is disrupted

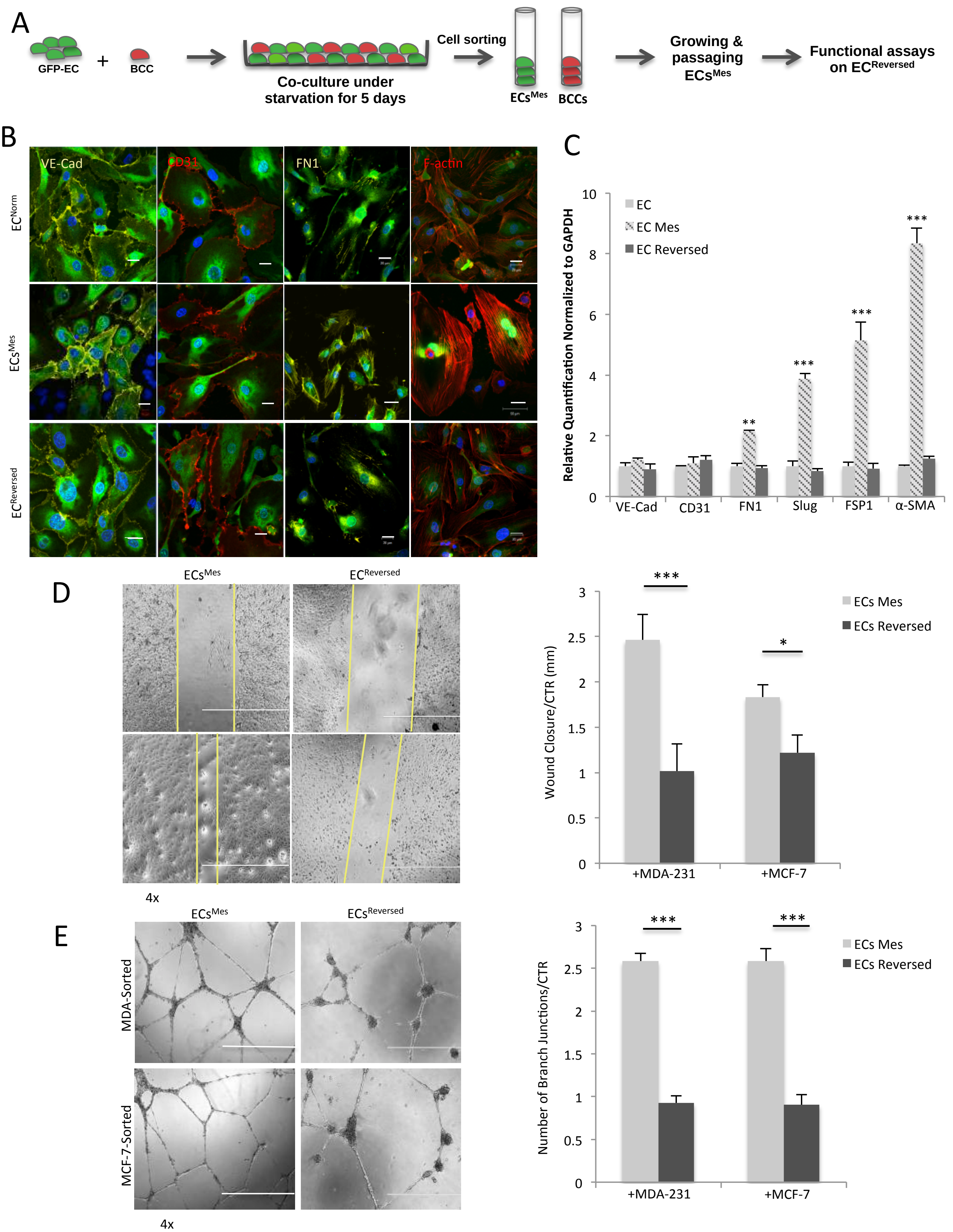

Figure S6. The EC mesenchymal property is reversed once their contact with cancer cells is disrupted

A) Schematic representation of the experimental procedure performed for obtaining $EC^{Reversed}$. ECs^{Mes} were sorted from BCCs and continued to grow and passage without any contact with BCCs under normal condition for 10-15 days to obtain $EC^{Reversed}$. These cells were then examined for the maintenance of mesenchymal phenotype in comparison to ECs^{Norm} and ECs^{Mes}.

B) Confocal microscopy images display the endothelial and mesenchymal properties of $EC^{Reversed}$ compared to ECs^{Norm} and ECs^{Mes}. As expected, endothelial properties were stable in all three conditions; however, the mesenchymal phenotype was reversed in $ECs^{Rverseed}$ 10-15 days after sorting from BCCs as indicated by down-regulation of mesenchymal marker FN1 and F-actin. Scale bars are representative of 20 µm.

C) Semi-quantitative qPCR analysis of endothelial and mesenchymal phenotypes in $EC^{Reversed}$ compared to ECs^{Norm} and ECs^{Mes}. In line with our confocal results, qPCR showed stable expression of CD31 and VE-Cad endothelial markers, while there was a reversion of mesenchymal phenotype in $EC^{Reversed}$ compared to ECs^{Mes}.

D) A wound healing assay performed to show and quantify the migratory capability of $EC^{Reversed}$ several passages post-sorting from MDA-231 tumor cells as compared with EC^{Mes}.

E) A tube formation assay evaluates and measures the angiogenic property of $EC^{Reversed}$ on matrigel. (***p<0.001, **p<0.01 and *p<0.05, mean ±SEM, n=3)

Figure S7. Proliferation of MCF-7 in co-culture with ECs^{Norm} or ECs^{Mes}.

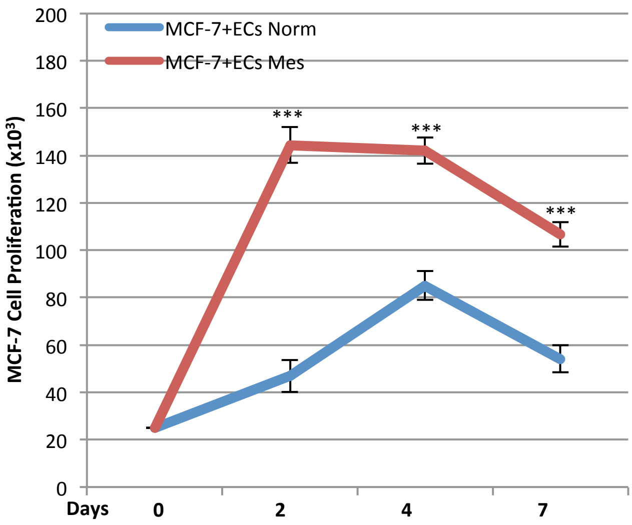

Cell proliferation assay showing proliferation of MCF-7 cancer cells when co-cultured with ECs^{Mes} or ECs^{Norm}. Once MCF-7 cells were grown with ECs^{Mes}, they showed around 1.6-fold higher proliferation rate than when grown with ECs^{Norm}.

Figure S8

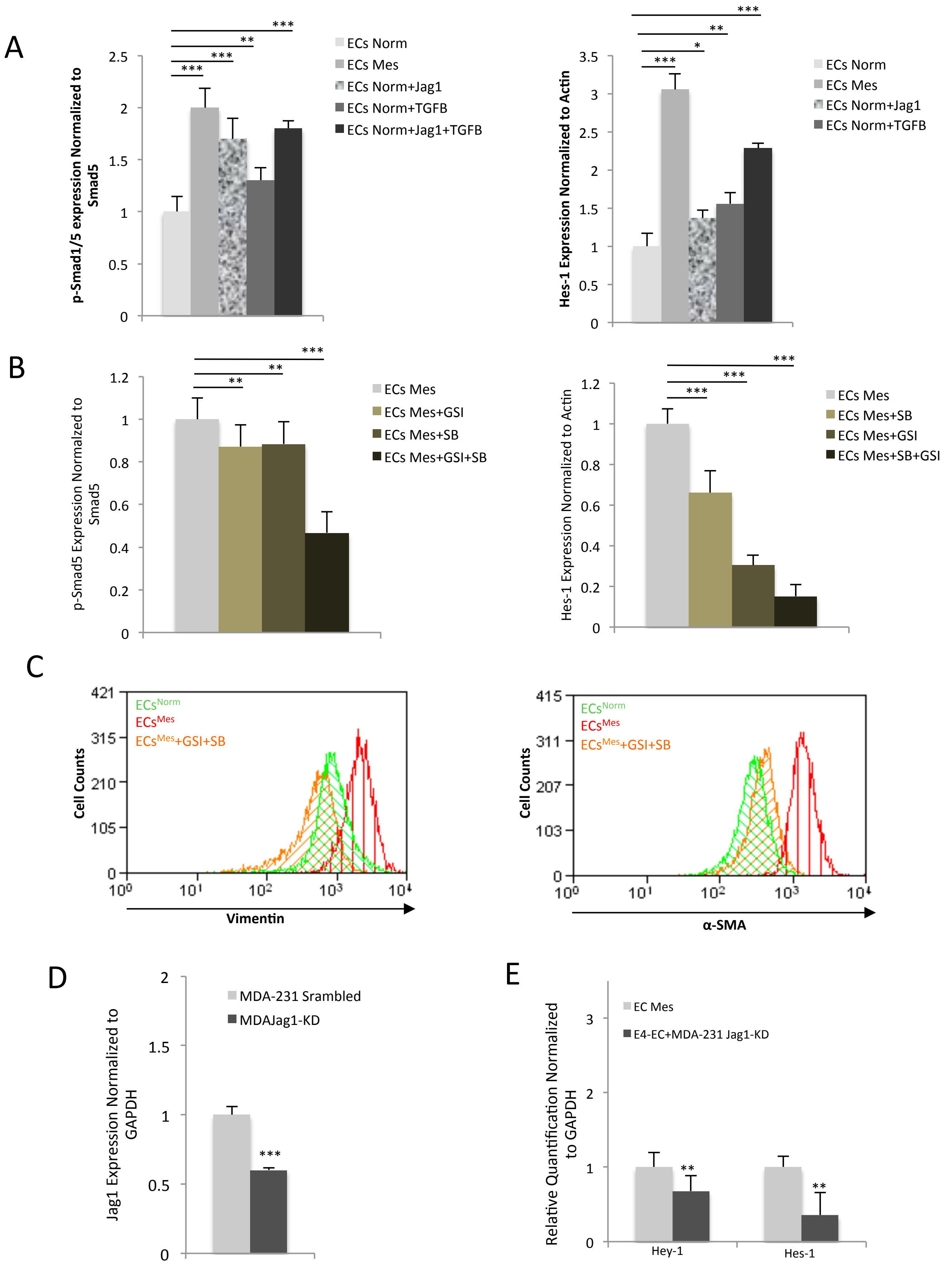

A
p-Smad1/5 expression Normalized to Smad5
2.5
2
1.5
1
0.5
0
***
**
***
***
ECs Norm
ECs Mes
ECs Norm+Jag1
ECs Norm+TGFB
ECs Norm+Jag1+TGFB
Hes-1 Expression Normalized to Actin
3.5
3
2.5
2
1.5
1
0.5
0
***
**
*
***
ECs Norm
ECs Mes
ECs Norm+Jag1
ECs Norm+TGFB
B
p-Smad5 Expression Normalzed to Smad5
1.2
1
0.8
0.6
0.4
0.2
0
***
**
**
ECs Mes
ECs Mes+GSI
ECs Mes+SB
ECs Mes+GSI+SB
Hes-1 Expression Normalized to Actin
1.2
1
0.8
0.6
0.4
0.2
0
***
***
***
ECs Mes
ECs Mes+SB
ECs Mes+GSI
ECs Mes+SB+GSI
C
421
315
210
105
0
Cell Counts
ECsNorm
ECsMes
ECsMes+GSI+SB
10⁰ 10¹ 10² 10³ 10⁴
Vimentin
415
311
207
103
0
Cell Counts
ECsNorm
ECsMes
ECsMes+GSI+SB
10⁰ 10¹ 10² 10³ 10⁴
α-SMA
D
Jag1 Expression Normalized to GAPDH
2
1.5
1
0.5
0
MDA-231 Srambled
MDAJag1-KD
***
E
Relative Quantification Normalized to GAPDH
3
2
1
0
EC Mes
E4-EC+MDA-231 Jag1-KD
**
**
Hey-1
Hes-1

Figure S8.

A) Western blot band densitometry showing the up-regulation of p-Smad5 in response to treating ECs^{Norm} with Jag1 or TGFβ or both ligands compared to total Smad5 protein (left bar graph). To show a synergy between Jag1 and TGFβ in activation of Smad5, we showed up-regulation of notch downstream effector Hes-1 once ECs^{Norm} were supplemented with the ligands (right bar graph). The highest level of Hes-1 activation is observed once ECs^{Norm} were treated with both ligands.

B) Western blot band densitometry demonstrating that inhibition of notch and/or TGFβ pathways by GSI and SB, respectively significantly down-regulated phosphorylation of Smad5 in ECs^{Mes} once ECs^{Mes} were treated with both inhibitors (left bar graph). This finding confirms the involvement of both pathways in Smad5 activation. Also, the lowest level of Hes-1 expression was found in ECs^{Mes} treated with both inhibitors (right bar graph) showing a synergy between the two pathways.

C) Flow cytometry overlays further confirming a role for TGFβ and notch pathways in regulation of tumor-induced mesenchymal transformation of ECs^{Mes}. Treating ECs^{Mes} with TGFβ and notch inhibitors resulted in down-regulation of mesenchymal markers such as α-SMA and vimentin in ECs^{Mes} as compared to ECs^{Norm} and ECs^{Mes} without inhibitors.

D) qPCR analysis of Jagged1 down-regulation in MDA-231 cells transfected with shRNA against Jag1.

E) qPCR analysis showing lack of activation of notch downstream effector molecules in ECs once they were exposed to $MDA\text{-}231^{Jag1\text{-}KD}$ cells further confirming Jag1 silencing on MDA-231 cells.

Supplementary Table S1. List of Primers

Primer Name	Forward	Reverse
FN1	CAGTGGGAGACCTCGAGAAG	TCCCTCGGAACATCAGAAAC
Snail	CCTCCCTGTCAGATGAGGAC	CCAGGCTGAGGTATTCCTTG
CD31	AAGTGGAGTCCAGCCGCATATC	ATGGAGCAGGACAGGTTCAGTC
VE-Cad	GAAGCCTCTGATTGGCACAGTG	TTTTGTGACTCGGAAGAACTGGC
Slug	GGGGAGAAGCCTTTTTCTTG	TCCTCATGTTTGTGCAGGAG
FSP1	GAGGCTTTACTCGCACTTCG	ACCCTACGCAGACTCCCAG
α-SMA	CTGACAGAGGCACCACTGAA	CATCTCCAGAGTCCAGCACA
Jag1	TCGCTGTATCTGTCCACCTG	AGTCACTGGCACGGTTGTA
Hey1	TGTCTGAGCTGAGAAGGCTGG	TTCAGGTGATCCACGGTCATC
Hes1	ATGGAGAAAAATTCCTCGTCC	TTCAGAGCATCCAAAATCAGT
GAPDH	AGCCACATCGCTCAGACAC	GCCCAATACGACCAAATCC
